# Supplementary material for: A viral SAVED protein with ring nuclease activity degrades the CRISPR second messenger cA4
Source: Biochem J. 2025 Nov 10;482(22):1707–19. doi: 10.1042/BCJ20253271 (PMC12751052; doi:10.1042/BCJ20253271)
Supplement: Online supplementary material 1 [file bcj-482-22-BCJ20253271-s001.pdf]

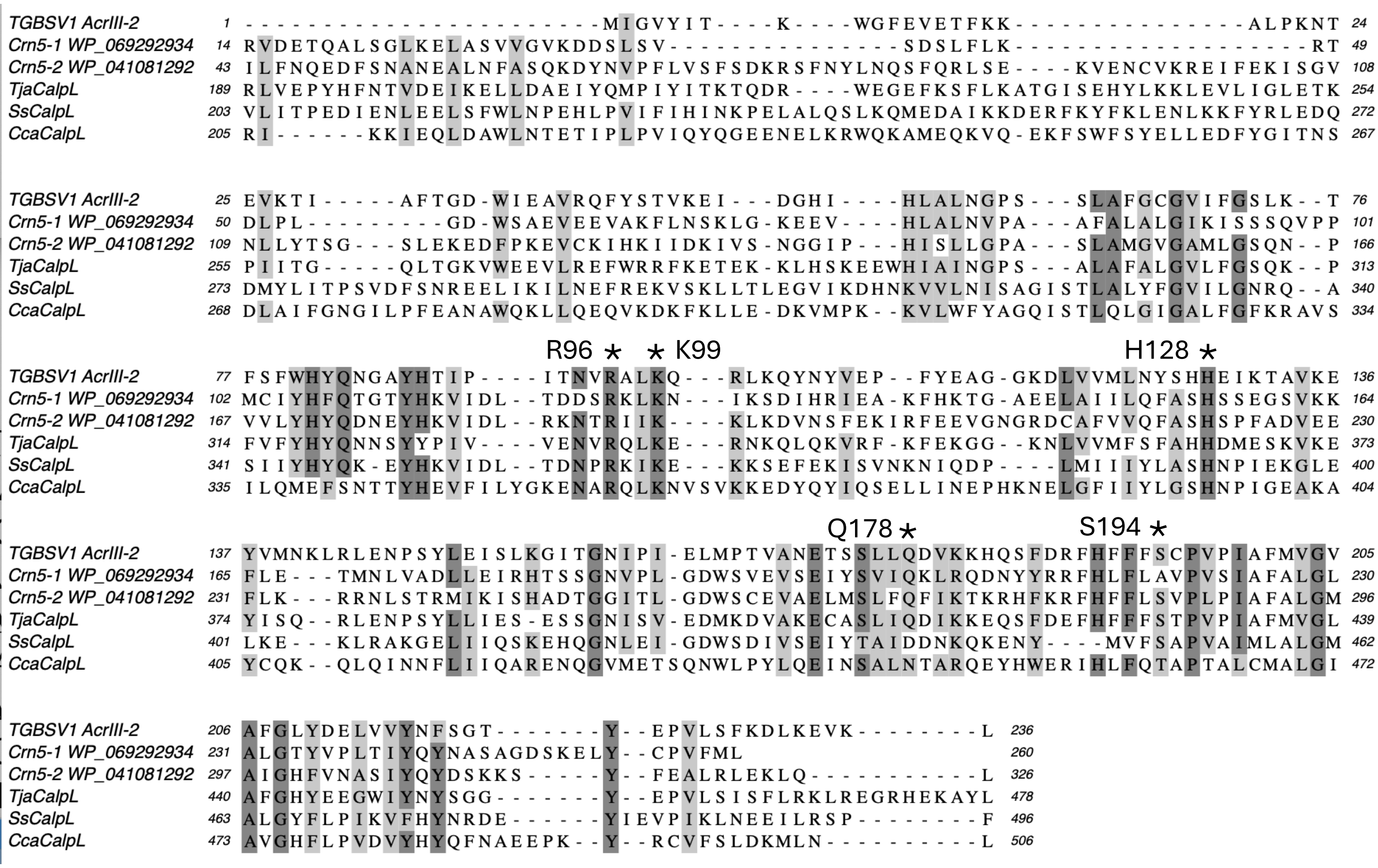

Supplementary Figure 1. Sequence alignment of the TGBSV SAVED domain protein AcrIII-2 with bacterial homologues. The CalpL proteins from *Thermocrinis jamiesonii* (Tja), *Sulfurihydrogenibium* spp (Ss) and*Candidatus* Cloacimonas acidaminovorans (Cca) are shown. Crn5-1 and 5-2 are presumed CRISPR ring nucleases. Residues investigated by site directed mutagenesis are labelled.

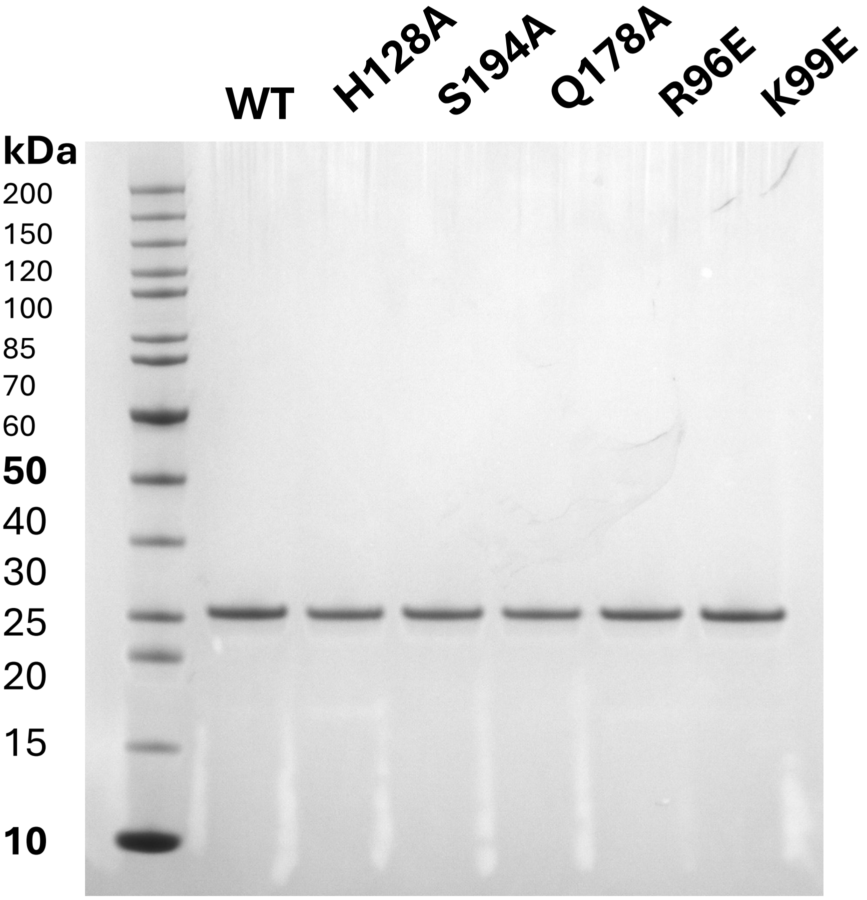

Supplementary Figure 2. Purified AcrIII-2 wild-type and variant proteins

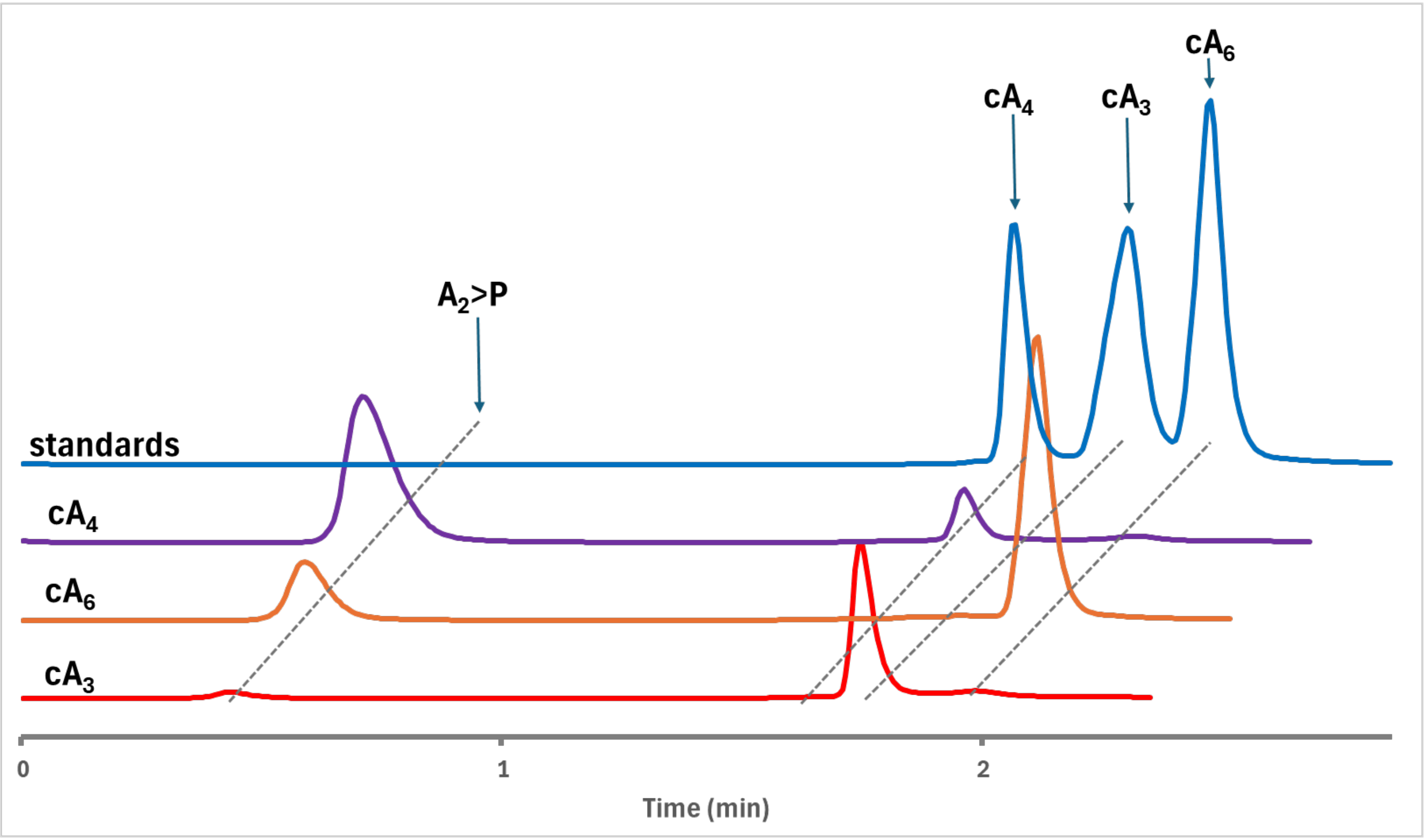

Supplementary Figure 3. AcrIII-2 is cA$_4$ specific. AcrIII-2 was incubated with cA$_3$, cA$_4$, and cA$_6$, respectively for 30 min at 60 ° C. AcrIII-2 primarily degrades cA$_4$, although cA$_6$ was degraded to a lesser extent. AcrIII-2 showed minimal ring nuclease activity with cA$_3$.

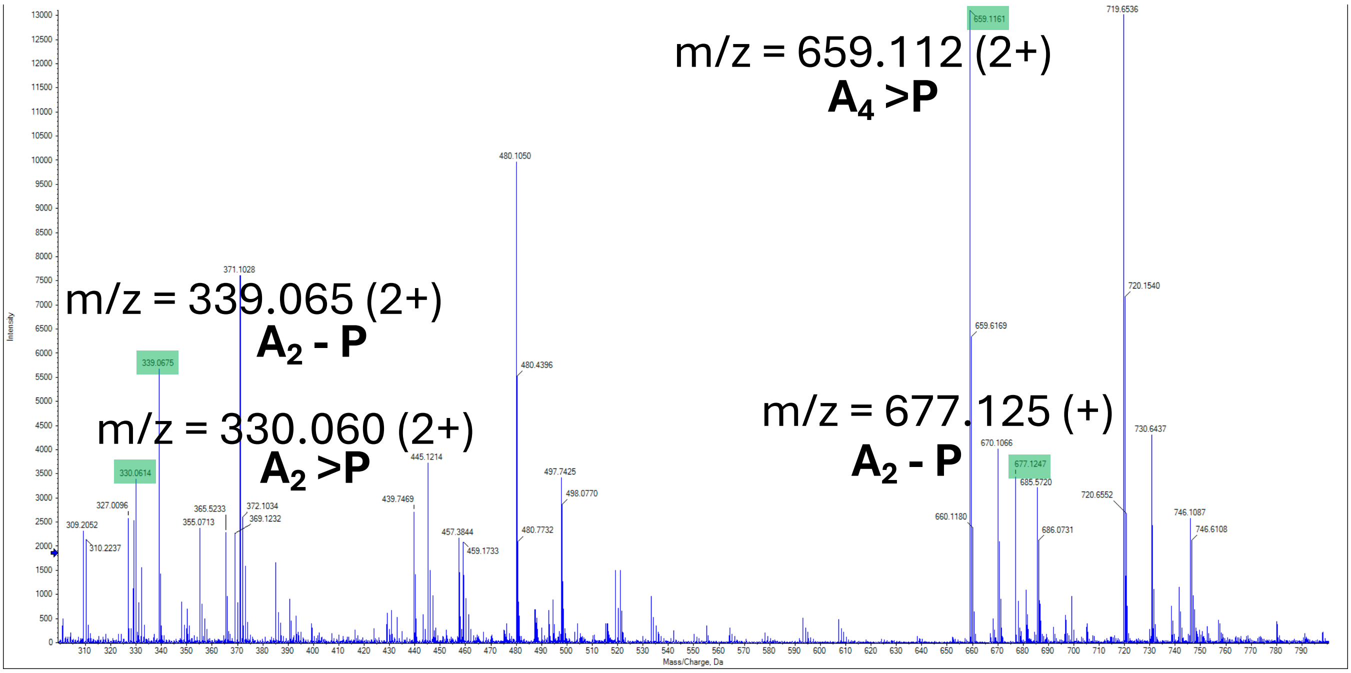

Supplementary Figure 4. Mass spectrometry analysis of the initial product of cA_4 degradation by AcrIII-2 (rightmost peak in Figure 1). This identifies A_4>P as the most likely initial product, as observed for the CalpL proteins.

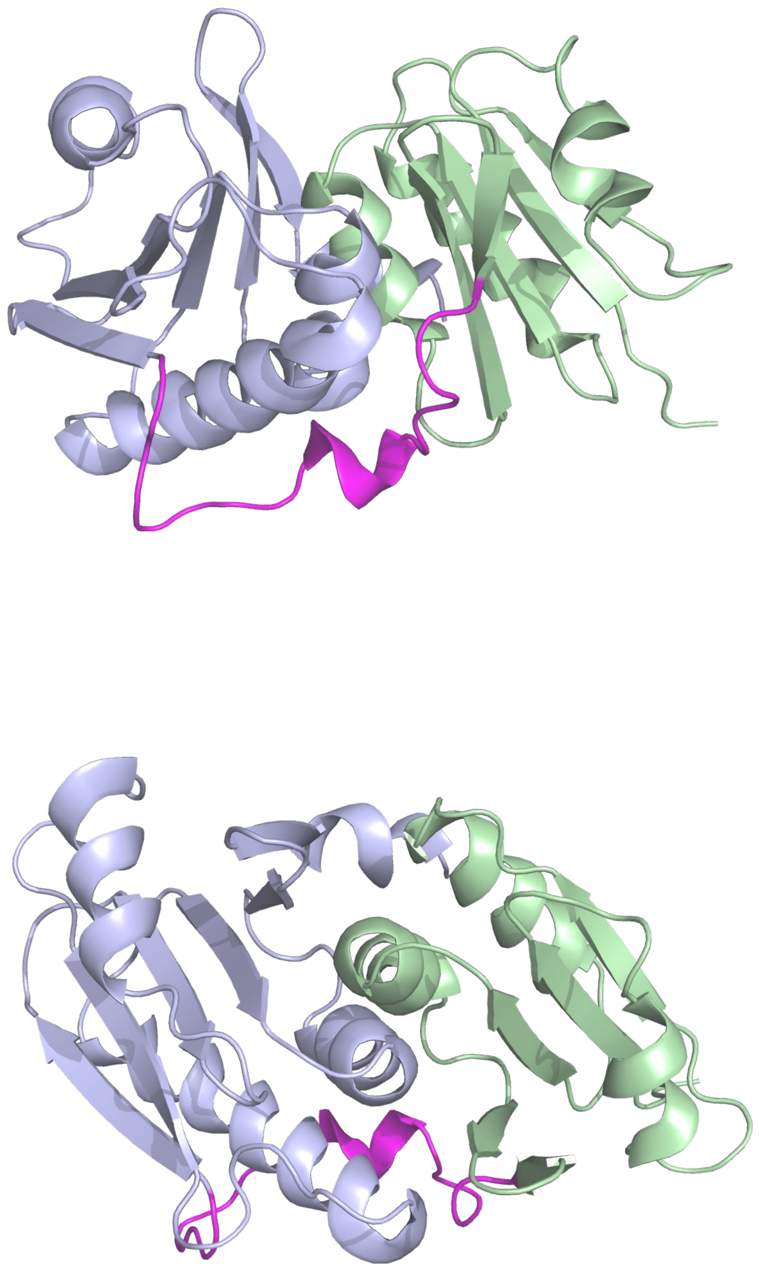

Supplementary Figure 5. Structure of the AcrIII-2 SAVED domain.
Two orthogonal views highlight the organization of the SAVED domain, which has two CARF domains (green and blue) joined by a linker peptide (magenta).

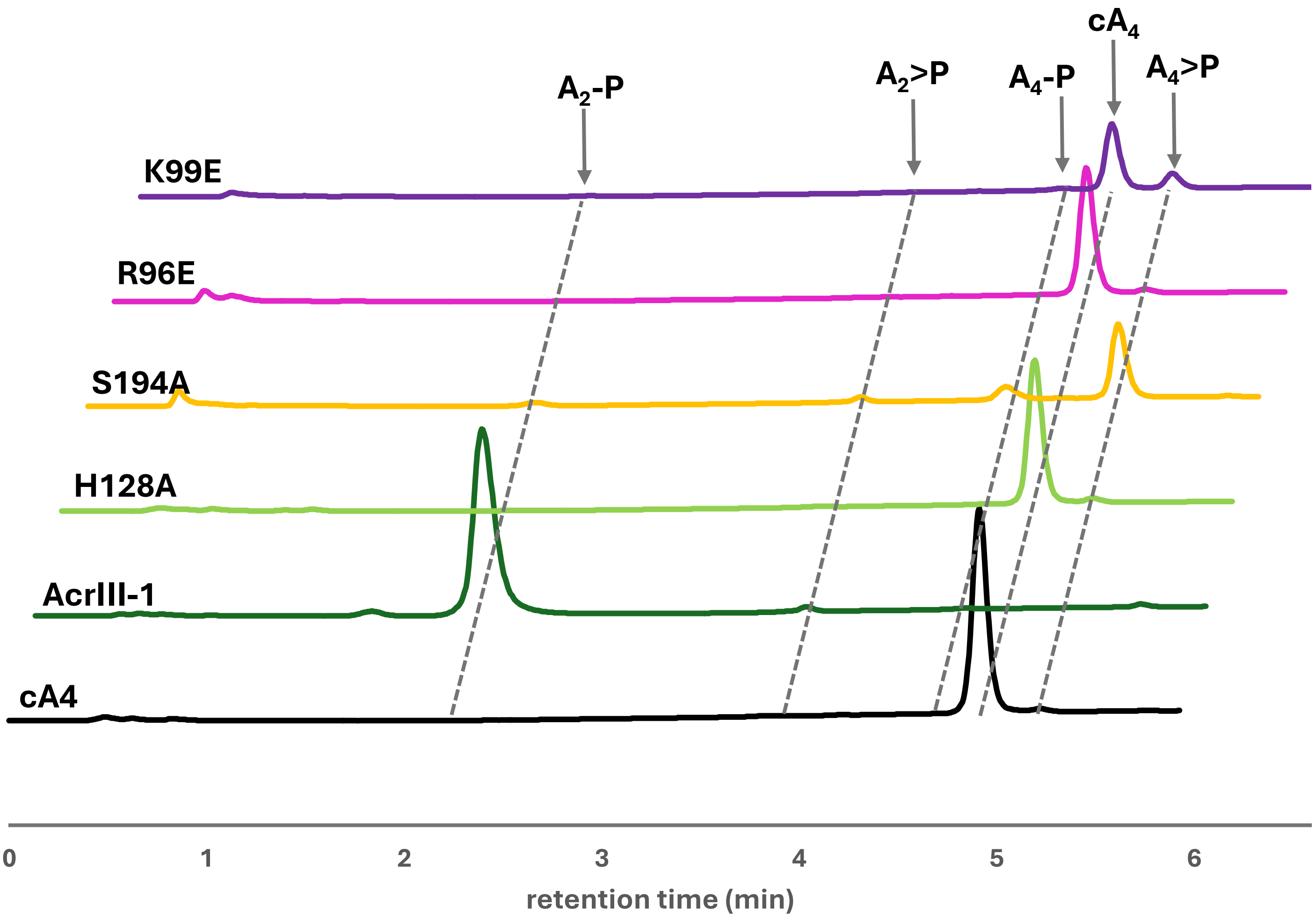

Supplementary Figure 6. Activity of mutant AcrIII-2 at 1:1 cA_4 to AcrIII-2. While H128A and R96E were still completely catalytically dead. K99E generated small amounts of A_4>P, and S194A completely converted cA_4 to linear tetranucleotide products.

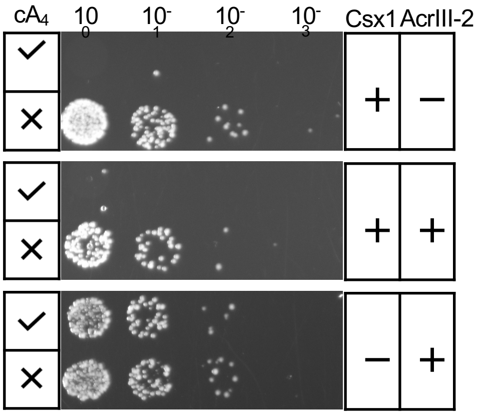

Supplementary Figure 7. Plasmid challenge assay with AcrIII-2.

Expression of AcrIII-2 did not affect colony forming unit (cfu) counts in cells with cA_4-activated Csx1. cA_4 was generated in the MtbCsm system programmed with a crRNA targeting the *tetR* gene, whereas no cA_4 was produced in the non-targeting (pUC) control system, indicated by ticks and crosses, respectively.

Supplementary Table 1: Data collection and refinement statistics for AcrIII-2

**Data Collection**	
Space group	P3121
Cell dimensions	
a, b, c (Å)	110.6, 110.6, 113.0
α, β, γ (°)	90, 90, 120
Resolution (Å) (high resolution)	95.79 – 1.79 (1.82 – 1.79)
R_{merge}	0.115 (4.572)
$I/\sigma(I)$	17.4 (0.4)
Completeness (%)	100 (100)
Aver. Redundancy	38.9 (29.6)
CC half	1.000 (0.345)
Vm ($Å^3$/Da)	3.71
Solvent (%)	66.9
**Refinement**	
Unique reflections	75593 (3754)
R_{work} / Rf_{ree}	16.5 / 20.7
Geometric deviations	
Bonds (Å) / Angles (°)	0.005 / 1.314
No. atoms (non H)	
Protein	3811
Water	204
Phosphate	10
B factors (Å)	
Protein	43.7
Water	44.1
Phosphate	46.2
Ramachandran	
Favored / outlier (%)	96.8 / 0.21
Molprobity score / centile	1.37 / 97

* Values in parentheses are for the highest-resolution shell.
